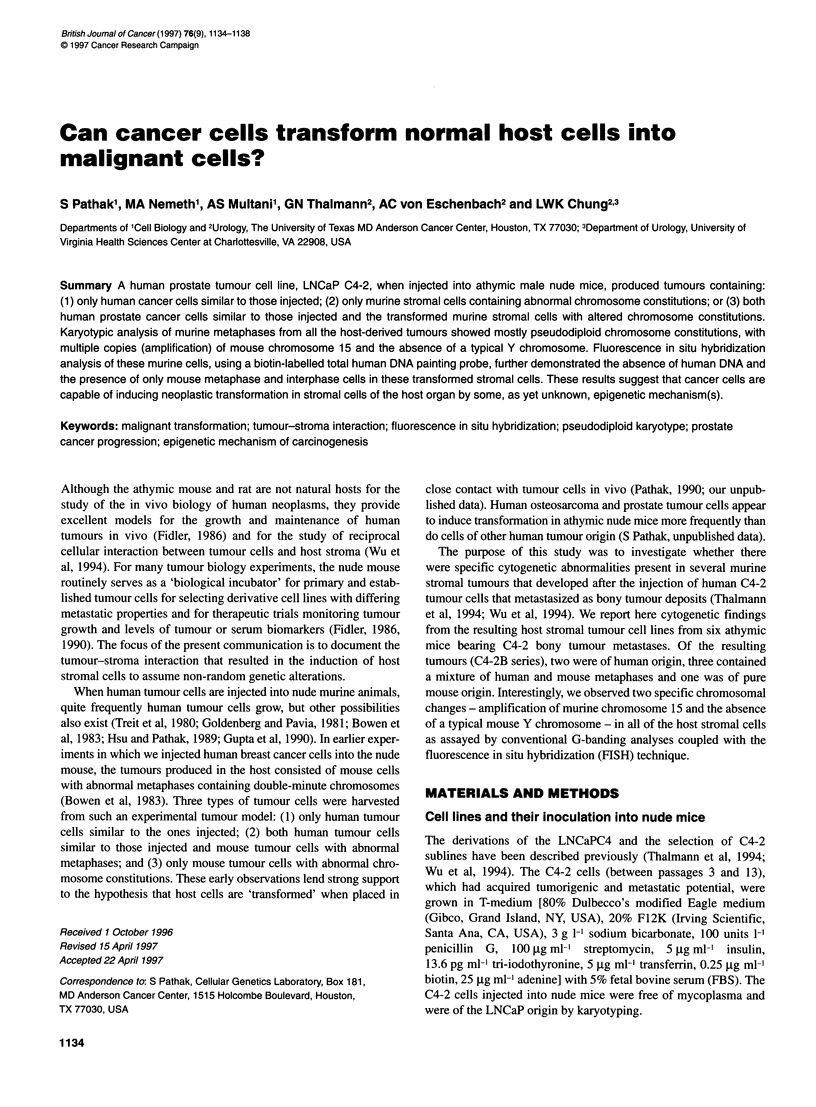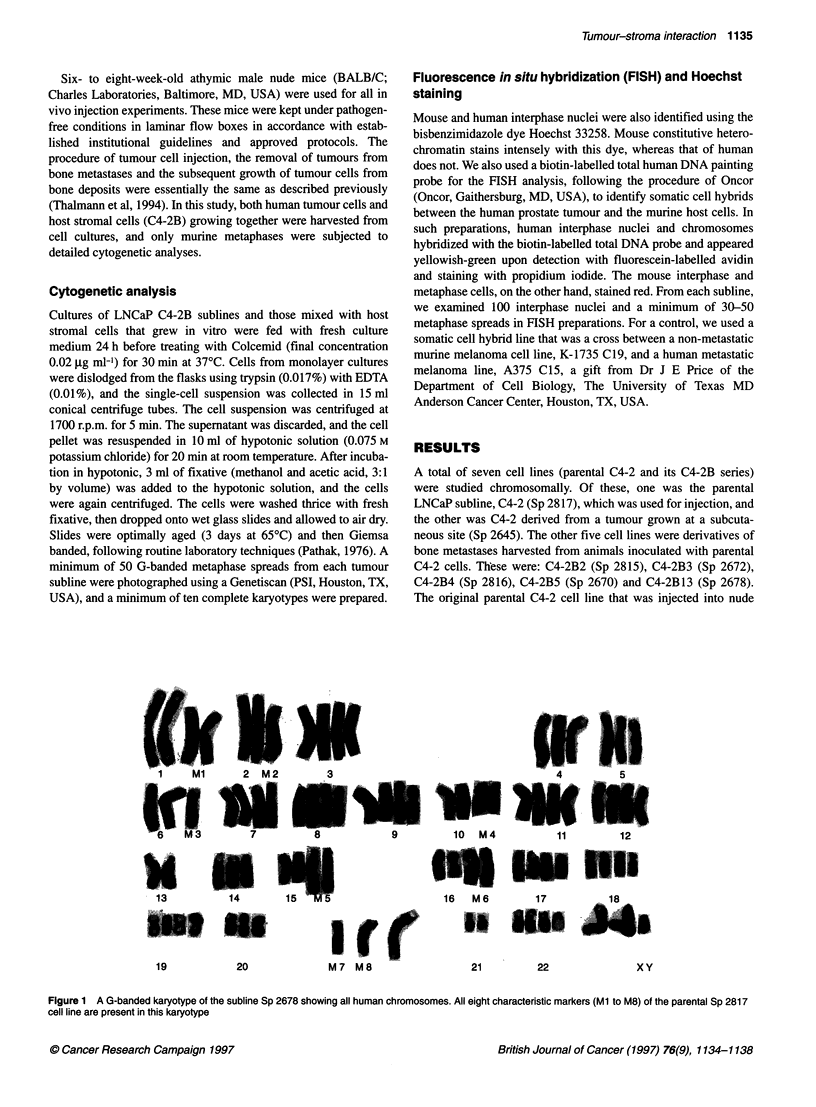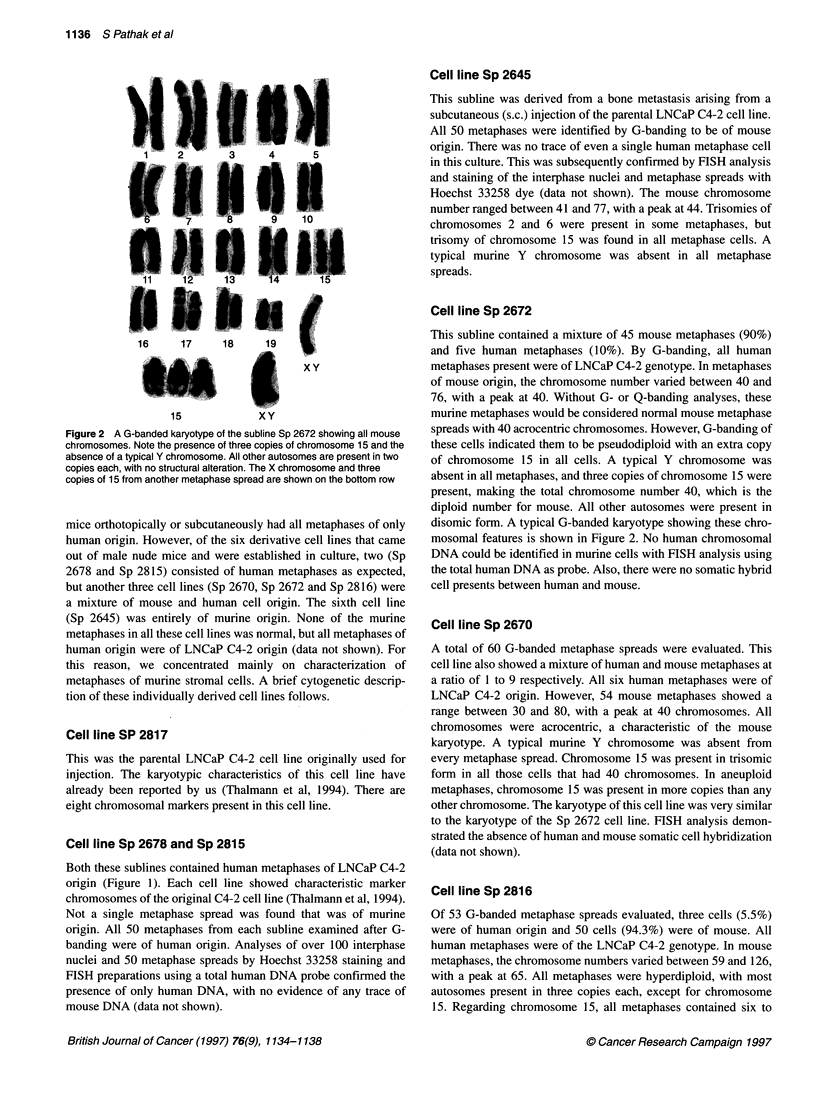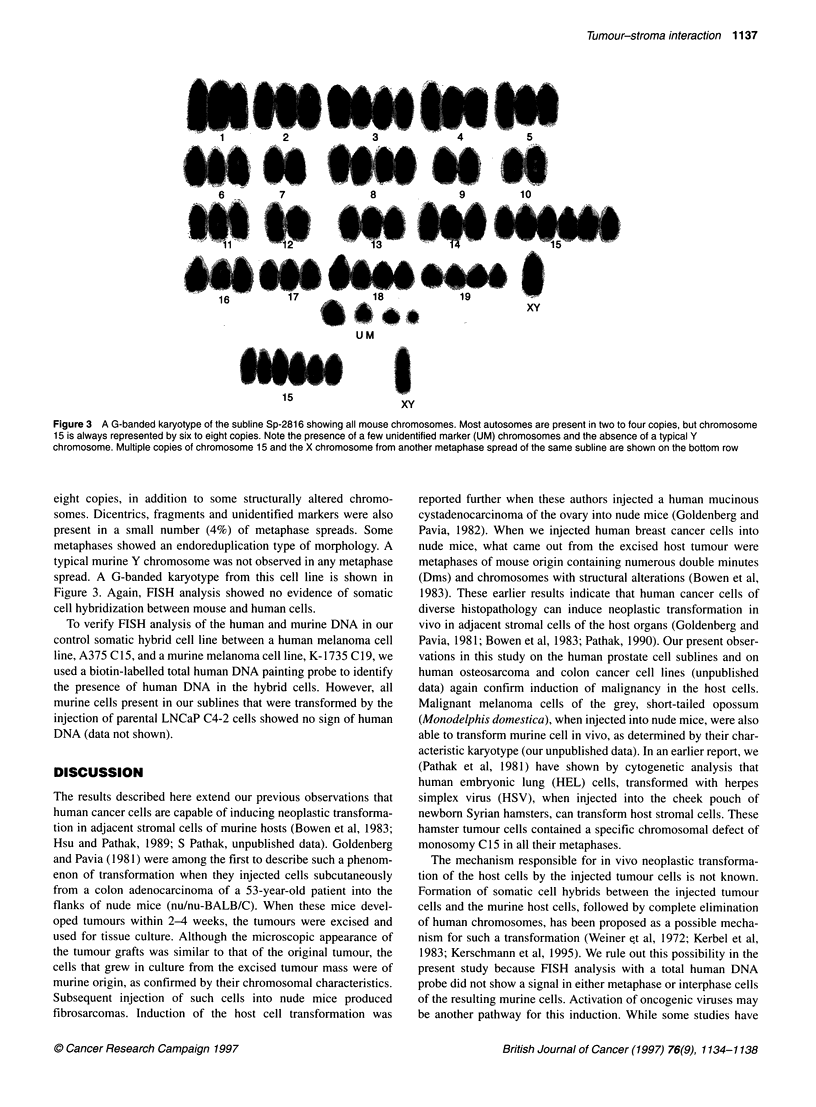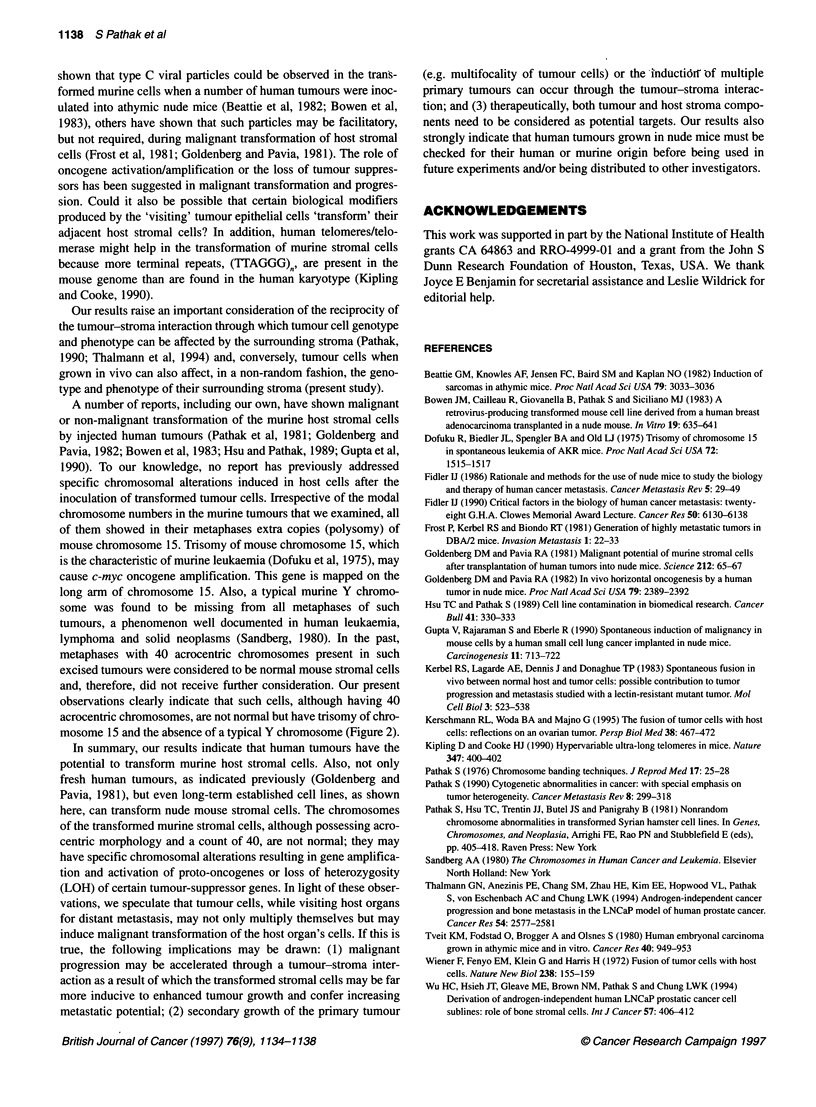# Can cancer cells transform normal host cells into malignant cells?

**DOI:** 10.1038/bjc.1997.524

**Published:** 1997

**Authors:** S. Pathak, M. A. Nemeth, A. S. Multani, G. N. Thalmann, A. C. von Eschenbach, L. W. Chung

**Affiliations:** Department of Cell Biology, The University of Texas MD Anderson Cancer Center, Houston 77030, USA.

## Abstract

**Images:**


British Journal of Cancer (1997) 76(9), 1134–1138
© 1997 Cancer Research Campaign

### Can cancer cells transform normal host cells into malignant cells?

**S Pathak[1], MA Nemeth[1], AS Multani[1], GN Thalmann[2], AC von Eschenbach[2] and LWK Chung[2,3]**

Departments of [1]Cell Biology and [2]Urology, The University of Texas MD Anderson Cancer Center, Houston, TX 77030; [3]Department of Urology, University of Virginia Health Sciences Center at Charlottesville, VA 22908, USA

**Summary** A human prostate tumour cell line, LNCaP C4-2, when injected into athymic male nude mice, produced tumours containing: (1) only human cancer cells similar to those injected; (2) only murine stromal cells containing abnormal chromosome constitutions; or (3) both human prostate cancer cells similar to those injected and the transformed murine stromal cells with altered chromosome constitutions. Karyotypic analysis of murine metaphases from all the host-derived tumours showed mostly pseudodiploid chromosome constitutions, with multiple copies (amplification) of mouse chromosome 15 and the absence of a typical Y chromosome. Fluorescence in situ hybridization analysis of these murine cells, using a biotin-labelled total human DNA painting probe, further demonstrated the absence of human DNA and the presence of only mouse metaphase and interphase cells in these transformed stromal cells. These results suggest that cancer cells are capable of inducing neoplastic transformation in stromal cells of the host organ by some, as yet unknown, epigenetic mechanism(s).

**Keywords:** malignant transformation; tumour–stroma interaction; fluorescence in situ hybridization; pseudodiploid karyotype; prostate cancer progression; epigenetic mechanism of carcinogenesis

Although the athymic mouse and rat are not natural hosts for the study of the in vivo biology of human neoplasms, they provide excellent models for the growth and maintenance of human tumours in vivo (Fidler, 1986) and for the study of reciprocal cellular interaction between tumour cells and host stroma (Wu et al, 1994). For many tumour biology experiments, the nude mouse routinely serves as a 'biological incubator' for primary and established tumour cells for selecting derivative cell lines with differing metastatic properties and for therapeutic trials monitoring tumour growth and levels of tumour or serum biomarkers (Fidler, 1986, 1990). The focus of the present communication is to document the tumour–stroma interaction that resulted in the induction of host stromal cells to assume non-random genetic alterations.

When human tumour cells are injected into nude murine animals, quite frequently human tumour cells grow, but other possibilities also exist (Treit et al, 1980; Goldenberg and Pavia, 1981; Bowen et al, 1983; Hsu and Pathak, 1989; Gupta et al, 1990). In earlier experiments in which we injected human breast cancer cells into the nude mouse, the tumours produced in the host consisted of mouse cells with abnormal metaphases containing double-minute chromosomes (Bowen et al, 1983). Three types of tumour cells were harvested from such an experimental tumour model: (1) only human tumour cells similar to the ones injected; (2) both human tumour cells similar to those injected and mouse tumour cells with abnormal metaphases; and (3) only mouse tumour cells with abnormal chromosome constitutions. These early observations lend strong support to the hypothesis that host cells are 'transformed' when placed in close contact with tumour cells in vivo (Pathak, 1990; our unpublished data). Human osteosarcoma and prostate tumour cells appear to induce transformation in athymic nude mice more frequently than do cells of other human tumour origin (S Pathak, unpublished data).

The purpose of this study was to investigate whether there were specific cytogenetic abnormalities present in several murine stromal tumours that developed after the injection of human C4-2 tumour cells that metastasized as bony tumour deposits (Thalmann et al, 1994; Wu et al, 1994). We report here cytogenetic findings from the resulting host stromal tumour cell lines from six athymic mice bearing C4-2 bony tumour metastases. Of the resulting tumours (C4-2B series), two were of human origin, three contained a mixture of human and mouse metaphases and one was of pure mouse origin. Interestingly, we observed two specific chromosomal changes – amplification of murine chromosome 15 and the absence of a typical mouse Y chromosome – in all of the host stromal cells as assayed by conventional G-banding analyses coupled with the fluorescence in situ hybridization (FISH) technique.

### MATERIALS AND METHODS

### Cell lines and their inoculation into nude mice

The derivations of the LNCaPC4 and the selection of C4-2 sublines have been described previously (Thalmann et al, 1994; Wu et al, 1994). The C4-2 cells (between passages 3 and 13), which had acquired tumorigenic and metastatic potential, were grown in T-medium [80% Dulbecco's modified Eagle medium (Gibco, Grand Island, NY, USA), 20% F12K (Irving Scientific, Santa Ana, CA, USA), 3 g $l^{-1}$ sodium bicarbonate, 100 units $l^{-1}$ penicillin G, 100 μg $ml^{-1}$ streptomycin, 5 μg $ml^{-1}$ insulin, 13.6 pg $ml^{-1}$ tri-iodothyronine, 5 μg $ml^{-1}$ transferrin, 0.25 μg $ml^{-1}$ biotin, 25 μg $ml^{-1}$ adenine] with 5% fetal bovine serum (FBS). The C4-2 cells injected into nude mice were free of mycoplasma and were of the LNCaP origin by karyotyping.

*Received 1 October 1996*
*Revised 15 April 1997*
*Accepted 22 April 1997*

*Correspondence to:* S Pathak, Cellular Genetics Laboratory, Box 181, MD Anderson Cancer Center, 1515 Holcombe Boulevard, Houston, TX 77030, USA

Six- to eight-week-old athymic male nude mice (BALB/C; Charles Laboratories, Baltimore, MD, USA) were used for all in vivo injection experiments. These mice were kept under pathogen-free conditions in laminar flow boxes in accordance with established institutional guidelines and approved protocols. The procedure of tumour cell injection, the removal of tumours from bone metastases and the subsequent growth of tumour cells from bone deposits were essentially the same as described previously (Thalmann et al, 1994). In this study, both human tumour cells and host stromal cells (C4-2B) growing together were harvested from cell cultures, and only murine metaphases were subjected to detailed cytogenetic analyses.

### Cytogenetic analysis

Cultures of LNCaP C4-2B sublines and those mixed with host stromal cells that grew in vitro were fed with fresh culture medium 24 h before treating with Colcemid (final concentration 0.02 μg $ml^{-1}$) for 30 min at 37°C. Cells from monolayer cultures were dislodged from the flasks using trypsin (0.017%) with EDTA (0.01%), and the single-cell suspension was collected in 15 ml conical centrifuge tubes. The cell suspension was centrifuged at 1700 r.p.m. for 5 min. The supernatant was discarded, and the cell pellet was resuspended in 10 ml of hypotonic solution (0.075 M potassium chloride) for 20 min at room temperature. After incubation in hypotonic, 3 ml of fixative (methanol and acetic acid, 3:1 by volume) was added to the hypotonic solution, and the cells were again centrifuged. The cells were washed thrice with fresh fixative, then dropped onto wet glass slides and allowed to air dry. Slides were optimally aged (3 days at 65°C) and then Giemsa banded, following routine laboratory techniques (Pathak, 1976). A minimum of 50 G-banded metaphase spreads from each tumour subline were photographed using a Genetiscan (PSI, Houston, TX, USA), and a minimum of ten complete karyotypes were prepared.

### Fluorescence *in situ* hybridization (FISH) and Hoechst staining

Mouse and human interphase nuclei were also identified using the bisbenzimidazole dye Hoechst 33258. Mouse constitutive heterochromatin stains intensely with this dye, whereas that of human does not. We also used a biotin-labelled total human DNA painting probe for the FISH analysis, following the procedure of Oncor (Oncor, Gaithersburg, MD, USA), to identify somatic cell hybrids between the human prostate tumour and the murine host cells. In such preparations, human interphase nuclei and chromosomes hybridized with the biotin-labelled total DNA probe and appeared yellowish-green upon detection with fluorescein-labelled avidin and staining with propidium iodide. The mouse interphase and metaphase cells, on the other hand, stained red. From each subline, we examined 100 interphase nuclei and a minimum of 30–50 metaphase spreads in FISH preparations. For a control, we used a somatic cell hybrid line that was a cross between a non-metastatic murine melanoma cell line, K-1735 C19, and a human metastatic melanoma line, A375 C15, a gift from Dr J E Price of the Department of Cell Biology, The University of Texas MD Anderson Cancer Center, Houston, TX, USA.

### RESULTS

A total of seven cell lines (parental C4-2 and its C4-2B series) were studied chromosomally. Of these, one was the parental LNCaP subline, C4-2 (Sp 2817), which was used for injection, and the other was C4-2 derived from a tumour grown at a subcutaneous site (Sp 2645). The other five cell lines were derivatives of bone metastases harvested from animals inoculated with parental C4-2 cells. These were: C4-2B2 (Sp 2815), C4-2B3 (Sp 2672), C4-2B4 (Sp 2816), C4-2B5 (Sp 2670) and C4-2B13 (Sp 2678). The original parental C4-2 cell line that was injected into nude

**Figure 1** A G-banded karyotype of the subline Sp 2678 showing all human chromosomes. All eight characteristic markers (M1 to M8) of the parental Sp 2817 cell line are present in this karyotype

**Figure 2** A G-banded karyotype of the subline Sp 2672 showing all mouse chromosomes. Note the presence of three copies of chromosome 15 and the absence of a typical Y chromosome. All other autosomes are present in two copies each, with no structural alteration. The X chromosome and three copies of 15 from another metaphase spread are shown on the bottom row mice orthotopically or subcutaneously had all metaphases of only human origin. However, of the six derivative cell lines that came out of male nude mice and were established in culture, two (Sp 2678 and Sp 2815) consisted of human metaphases as expected, but another three cell lines (Sp 2670, Sp 2672 and Sp 2816) were a mixture of mouse and human cell origin. The sixth cell line (Sp 2645) was entirely of murine origin. None of the murine metaphases in all these cell lines was normal, but all metaphases of human origin were of LNCaP C4-2 origin (data not shown). For this reason, we concentrated mainly on characterization of metaphases of murine stromal cells. A brief cytogenetic description of these individually derived cell lines follows.

### Cell line SP 2817

This was the parental LNCaP C4-2 cell line originally used for injection. The karyotypic characteristics of this cell line have already been reported by us (Thalmann et al, 1994). There are eight chromosomal markers present in this cell line.

### Cell line Sp 2678 and Sp 2815

Both these sublines contained human metaphases of LNCaP C4-2 origin (Figure 1). Each cell line showed characteristic marker chromosomes of the original C4-2 cell line (Thalmann et al, 1994). Not a single metaphase spread was found that was of murine origin. All 50 metaphases from each subline examined after G-banding were of human origin. Analyses of over 100 interphase nuclei and 50 metaphase spreads by Hoechst 33258 staining and FISH preparations using a total human DNA probe confirmed the presence of only human DNA, with no evidence of any trace of mouse DNA (data not shown).

### Cell line Sp 2645

This subline was derived from a bone metastasis arising from a subcutaneous (s.c.) injection of the parental LNCaP C4-2 cell line. All 50 metaphases were identified by G-banding to be of mouse origin. There was no trace of even a single human metaphase cell in this culture. This was subsequently confirmed by FISH analysis and staining of the interphase nuclei and metaphase spreads with Hoechst 33258 dye (data not shown). The mouse chromosome number ranged between 41 and 77, with a peak at 44. Trisomies of chromosomes 2 and 6 were present in some metaphases, but trisomy of chromosome 15 was found in all metaphase cells. A typical murine Y chromosome was absent in all metaphase spreads.

### Cell line Sp 2672

This subline contained a mixture of 45 mouse metaphases (90%) and five human metaphases (10%). By G-banding, all human metaphases present were of LNCaP C4-2 genotype. In metaphases of mouse origin, the chromosome number varied between 40 and 76, with a peak at 40. Without G- or Q-banding analyses, these murine metaphases would be considered normal mouse metaphase spreads with 40 acrocentric chromosomes. However, G-banding of these cells indicated them to be pseudodiploid with an extra copy of chromosome 15 in all cells. A typical Y chromosome was absent in all metaphases, and three copies of chromosome 15 were present, making the total chromosome number 40, which is the diploid number for mouse. All other autosomes were present in disomic form. A typical G-banded karyotype showing these chromosomal features is shown in Figure 2. No human chromosomal DNA could be identified in murine cells with FISH analysis using the total human DNA as probe. Also, there were no somatic hybrid cell presents between human and mouse.

### Cell line Sp 2670

A total of 60 G-banded metaphase spreads were evaluated. This cell line also showed a mixture of human and mouse metaphases at a ratio of 1 to 9 respectively. All six human metaphases were of LNCaP C4-2 origin. However, 54 mouse metaphases showed a range between 30 and 80, with a peak at 40 chromosomes. All chromosomes were acrocentric, a characteristic of the mouse karyotype. A typical murine Y chromosome was absent from every metaphase spread. Chromosome 15 was present in trisomic form in all those cells that had 40 chromosomes. In aneuploid metaphases, chromosome 15 was present in more copies than any other chromosome. The karyotype of this cell line was very similar to the karyotype of the Sp 2672 cell line. FISH analysis demonstrated the absence of human and mouse somatic cell hybridization (data not shown).

### Cell line Sp 2816

Of 53 G-banded metaphase spreads evaluated, three cells (5.5%) were of human origin and 50 cells (94.3%) were of mouse. All human metaphases were of the LNCaP C4-2 genotype. In mouse metaphases, the chromosome numbers varied between 59 and 126, with a peak at 65. All metaphases were hyperdiploid, with most autosomes present in three copies each, except for chromosome 15. Regarding chromosome 15, all metaphases contained six to

 © Cancer Research Campaign 1997

**Figure 3** A G-banded karyotype of the subline Sp-2816 showing all mouse chromosomes. Most autosomes are present in two to four copies, but chromosome 15 is always represented by six to eight copies. Note the presence of a few unidentified marker (UM) chromosomes and the absence of a typical Y chromosome. Multiple copies of chromosome 15 and the X chromosome from another metaphase spread of the same subline are shown on the bottom row eight copies, in addition to some structurally altered chromosomes. Dicentrics, fragments and unidentified markers were also present in a small number (4%) of metaphase spreads. Some metaphases showed an endoreduplication type of morphology. A typical murine Y chromosome was not observed in any metaphase spread. A G-banded karyotype from this cell line is shown in Figure 3. Again, FISH analysis showed no evidence of somatic cell hybridization between mouse and human cells.

To verify FISH analysis of the human and murine DNA in our control somatic hybrid cell line between a human melanoma cell line, A375 C15, and a murine melanoma cell line, K-1735 C19, we used a biotin-labelled total human DNA painting probe to identify the presence of human DNA in the hybrid cells. However, all murine cells present in our sublines that were transformed by the injection of parental LNCaP C4-2 cells showed no sign of human DNA (data not shown).

### DISCUSSION

The results described here extend our previous observations that human cancer cells are capable of inducing neoplastic transformation in adjacent stromal cells of murine hosts (Bowen et al, 1983; Hsu and Pathak, 1989; S Pathak, unpublished data). Goldenberg and Pavia (1981) were among the first to describe such a phenomenon of transformation when they injected cells subcutaneously from a colon adenocarcinoma of a 53-year-old patient into the flanks of nude mice (nu/nu-BALB/C). When these mice developed tumours within 2–4 weeks, the tumours were excised and used for tissue culture. Although the microscopic appearance of the tumour grafts was similar to that of the original tumour, the cells that grew in culture from the excised tumour mass were of murine origin, as confirmed by their chromosomal characteristics. Subsequent injection of such cells into nude mice produced fibrosarcomas. Induction of the host cell transformation was reported further when these authors injected a human mucinous cystadenocarcinoma of the ovary into nude mice (Goldenberg and Pavia, 1982). When we injected human breast cancer cells into nude mice, what came out from the excised host tumour were metaphases of mouse origin containing numerous double minutes (Dms) and chromosomes with structural alterations (Bowen et al, 1983). These earlier results indicate that human cancer cells of diverse histopathology can induce neoplastic transformation in vivo in adjacent stromal cells of the host organs (Goldenberg and Pavia, 1981; Bowen et al, 1983; Pathak, 1990). Our present observations in this study on the human prostate cell sublines and on human osteosarcoma and colon cancer cell lines (unpublished data) again confirm induction of malignancy in the host cells. Malignant melanoma cells of the grey, short-tailed opossum (*Monodelphis domestica*), when injected into nude mice, were also able to transform murine cell in vivo, as determined by their characteristic karyotype (our unpublished data). In an earlier report, we (Pathak et al, 1981) have shown by cytogenetic analysis that human embryonic lung (HEL) cells, transformed with herpes simplex virus (HSV), when injected into the cheek pouch of newborn Syrian hamsters, can transform host stromal cells. These hamster tumour cells contained a specific chromosomal defect of monosomy C15 in all their metaphases.

The mechanism responsible for in vivo neoplastic transformation of the host cells by the injected tumour cells is not known. Formation of somatic cell hybrids between the injected tumour cells and the murine host cells, followed by complete elimination of human chromosomes, has been proposed as a possible mechanism for such a transformation (Weiner et al, 1972; Kerbel et al, 1983; Kerschmann et al, 1995). We rule out this possibility in the present study because FISH analysis with a total human DNA probe did not show a signal in either metaphase or interphase cells of the resulting murine cells. Activation of oncogenic viruses may be another pathway for this induction. While some studies have

© Cancer Research Campaign 1997 

shown that type C viral particles could be observed in the transformed murine cells when a number of human tumours were inoculated into athymic nude mice (Beattie et al, 1982; Bowen et al, 1983), others have shown that such particles may be facilitatory, but not required, during malignant transformation of host stromal cells (Frost et al, 1981; Goldenberg and Pavia, 1981). The role of oncogene activation/amplification or the loss of tumour suppressors has been suggested in malignant transformation and progression. Could it also be possible that certain biological modifiers produced by the 'visiting' tumour epithelial cells 'transform' their adjacent host stromal cells? In addition, human telomeres/telomerase might help in the transformation of murine stromal cells because more terminal repeats, $(TTAGGG)_n$, are present in the mouse genome than are found in the human karyotype (Kipling and Cooke, 1990).

Our results raise an important consideration of the reciprocity of the tumour–stroma interaction through which tumour cell genotype and phenotype can be affected by the surrounding stroma (Pathak, 1990; Thalmann et al, 1994) and, conversely, tumour cells when grown in vivo can also affect, in a non-random fashion, the genotype and phenotype of their surrounding stroma (present study).

A number of reports, including our own, have shown malignant or non-malignant transformation of the murine host stromal cells by injected human tumours (Pathak et al, 1981; Goldenberg and Pavia, 1982; Bowen et al, 1983; Hsu and Pathak, 1989; Gupta et al, 1990). To our knowledge, no report has previously addressed specific chromosomal alterations induced in host cells after the inoculation of transformed tumour cells. Irrespective of the modal chromosome numbers in the murine tumours that we examined, all of them showed in their metaphases extra copies (polysomy) of mouse chromosome 15. Trisomy of mouse chromosome 15, which is the characteristic of murine leukaemia (Dofuku et al, 1975), may cause *c-myc* oncogene amplification. This gene is mapped on the long arm of chromosome 15. Also, a typical murine Y chromosome was found to be missing from all metaphases of such tumours, a phenomenon well documented in human leukaemia, lymphoma and solid neoplasms (Sandberg, 1980). In the past, metaphases with 40 acrocentric chromosomes present in such excised tumours were considered to be normal mouse stromal cells and, therefore, did not receive further consideration. Our present observations clearly indicate that such cells, although having 40 acrocentric chromosomes, are not normal but have trisomy of chromosome 15 and the absence of a typical Y chromosome (Figure 2).

In summary, our results indicate that human tumours have the potential to transform murine host stromal cells. Also, not only fresh human tumours, as indicated previously (Goldenberg and Pavia, 1981), but even long-term established cell lines, as shown here, can transform nude mouse stromal cells. The chromosomes of the transformed murine stromal cells, although possessing acrocentric morphology and a count of 40, are not normal; they may have specific chromosomal alterations resulting in gene amplification and activation of proto-oncogenes or loss of heterozygosity (LOH) of certain tumour-suppressor genes. In light of these observations, we speculate that tumour cells, while visiting host organs for distant metastasis, may not only multiply themselves but may induce malignant transformation of the host organ's cells. If this is true, the following implications may be drawn: (1) malignant progression may be accelerated through a tumour–stroma interaction as a result of which the transformed stromal cells may be far more inducive to enhanced tumour growth and confer increasing metastatic potential; (2) secondary growth of the primary tumour (e.g. multifocality of tumour cells) or the induction of multiple primary tumours can occur through the tumour–stroma interaction; and (3) therapeutically, both tumour and host stroma components need to be considered as potential targets. Our results also strongly indicate that human tumours grown in nude mice must be checked for their human or murine origin before being used in future experiments and/or being distributed to other investigators.

### ACKNOWLEDGEMENTS

This work was supported in part by the National Institute of Health grants CA 64863 and RRO-4999-01 and a grant from the John S Dunn Research Foundation of Houston, Texas, USA. We thank Joyce E Benjamin for secretarial assistance and Leslie Wildrick for editorial help.

### REFERENCES

Beattie GM, Knowles AF, Jensen FC, Baird SM and Kaplan NO (1982) Induction of sarcomas in athymic mice. *Proc Natl Acad Sci USA* **79**: 3033–3036

Bowen JM, Cailleau R, Giovanella B, Pathak S and Siciliano MJ (1983) A retrovirus-producing transformed mouse cell line derived from a human breast adenocarcinoma transplanted in a nude mouse. *In Vitro* **19**: 635–641

Dofuku R, Biedler JL, Spengler BA and Old LJ (1975) Trisomy of chromosome 15 in spontaneous leukemia of AKR mice. *Proc Natl Acad Sci USA* **72**: 1515–1517

Fidler IJ (1986) Rationale and methods for the use of nude mice to study the biology and therapy of human cancer metastasis. *Cancer Metastasis Rev* **5**: 29–49

Fidler IJ (1990) Critical factors in the biology of human cancer metastasis: twenty-eight G.H.A. Clowes Memorial Award Lecture. *Cancer Res* **50**: 6130–6138

Frost P, Kerbel RS and Biondo RT (1981) Generation of highly metastatic tumors in DBA/2 mice. *Invasion Metastasis* **1**: 22–33

Goldenberg DM and Pavia RA (1981) Malignant potential of murine stromal cells after transplantation of human tumors into nude mice. *Science* **212**: 65–67

Goldenberg DM and Pavia RA (1982) In vivo horizontal oncogenesis by a human tumor in nude mice. *Proc Natl Acad Sci USA* **79**: 2389–2392

Hsu TC and Pathak S (1989) Cell line contamination in biomedical research. *Cancer Bull* **41**: 330–333

Gupta V, Rajaraman S and Eberle R (1990) Spontaneous induction of malignancy in mouse cells by a human small cell lung cancer implanted in nude mice. *Carcinogenesis* **11**: 713–722

Kerbel RS, Lagarde AE, Dennis J and Donaghue TP (1983) Spontaneous fusion in vivo between normal host and tumor cells: possible contribution to tumor progression and metastasis studied with a lectin-resistant mutant tumor. *Mol Cell Biol* **3**: 523–538

Kerschmann RL, Woda BA and Majno G (1995) The fusion of tumor cells with host cells: reflections on an ovarian tumor. *Persp Biol Med* **38**: 467–472

Kipling D and Cooke HJ (1990) Hypervariable ultra-long telomeres in mice. *Nature* **347**: 400–402

Pathak S (1976) Chromosome banding techniques. *J Reprod Med* **17**: 25–28

Pathak S (1990) Cytogenetic abnormalities in cancer: with special emphasis on tumor heterogeneity. *Cancer Metastasis Rev* **8**: 299–318

Pathak S, Hsu TC, Trentin JJ, Butel JS and Panigrahy B (1981) Nonrandom chromosome abnormalities in transformed Syrian hamster cell lines. In *Genes, Chromosomes, and Neoplasia*, Arrighi FE, Rao PN and Stubblefield E (eds), pp. 405–418. Raven Press: New York

Sandberg AA (1980) *The Chromosomes in Human Cancer and Leukemia*. Elsevier North Holland: New York

Thalmann GN, Anezinis PE, Chang SM, Zhau HE, Kim EE, Hopwood VL, Pathak S, von Eschenbach AC and Chung LWK (1994) Androgen-independent cancer progression and bone metastasis in the LNCaP model of human prostate cancer. *Cancer Res* **54**: 2577–2581

Tveit KM, Fodstad O, Brogger A and Olsnes S (1980) Human embryonal carcinoma grown in athymic mice and in vitro. *Cancer Res* **40**: 949–953

Wiener F, Fenyo EM, Klein G and Harris H (1972) Fusion of tumor cells with host cells. *Nature New Biol* **238**: 155–159

Wu HC, Hsieh JT, Gleave ME, Brown NM, Pathak S and Chung LWK (1994) Derivation of androgen-independent human LNCaP prostatic cancer cell sublines: role of bone stromal cells. *Int J Cancer* **57**: 406–412

© Cancer Research Campaign 1997